# Assessing the impacts of extreme precipitation on groundwater recharge under diverse hydrogeological conditions via the theis equation

**DOI:** 10.1016/j.isci.2026.115691

**Published:** 2026-04-14

**Authors:** Qingtao Ma, Xinghang Zhang, Zhijie Bai, Sishan Pang, Guofei Shang, Zhaowang Chen, Ruohan Chen, Xiaoxia Hou, Yue Liu, Zhixian Wang, Shumin Han, Dandan Ren, Xiaojing Liu, Zhitong Ma

**Affiliations:** 1 School of Land Science and Space Planning/Hebei International Joint Research Center for Remote Sensing of Agricultural Drought Monitoring, Hebei GEO University, Shijiazhuang 050031, China; 2 Satellite Application Center for Ecology and Environment, Ministry of Ecology and Environment, Beijing 100094, China; 3 College of Life Sciences, Hebei University, Baoding 071002, China; 4 The Third Geological Team of Hebei Provincial Bureau of Geology and Mineral Resources Exploration and Development, Zhangjiakou City, Hebei Province, China; 5 Center for Agricultural Resources Research, Institute of Genetics and Developmental Biology, Chinese Academy of Sciences, Shijiazhuang 050022, China; 6 School of Resources and Environment (College of Carbon Neutrality), Linyi University, Linyi, China; 7 Center for Geophysical Survey, China Geological Survey, Lang Fang 065000, China; 8 College of Geology and Environment, Xi’an University of Science and Technology, Xi’an 710054, China

**Keywords:** Meteorology, Environmental science, Hydrology

## Abstract

Climate change has amplified the frequency of extreme high-intensity precipitation events, altering groundwater recharge across diverse hydrogeological settings. Using observed precipitation, groundwater level (GL), hydrogeological drilling data, and the Theis equation, we quantified how precipitation affects groundwater in the Baiyangdian-Basin Plain. We found that GL declined in 80% of counties, with a milder decrease in piedmont regions. During rainy and irrigation periods, the cumulative rise and fall of GL accounted for 55.3% and 57.5% of the annual total, respectively. Short-duration intense precipitation dominates annual water gain in wet years and amplifies inter- and intra-annual GL fluctuations. When precipitation exceeds 600 mm/year (intensity >8 mm/day), both GL recovery and well flow rate decline rapidly, implying reduced groundwater recharge. In the piedmont, precipitation is strongly positively correlated with GL and well flow rate, indicating little impact of extreme precipitation on groundwater recharge. These findings prevent the overestimation of groundwater recharge from extreme precipitation.

## Highlights

• Increased precipitation in Baiyangdian Basin is mainly from extreme heavy rain
• Extreme precipitation drives GL fluctuation and decline in wet irrigation seasons
• GL recovery and well flowrate decline sharply when precipitation exceeds 600mm/yr or 8mm/d
• Piedmont groundwater recharge is less affected by extreme rain than inland

## Graphical abstract

**Figure:**
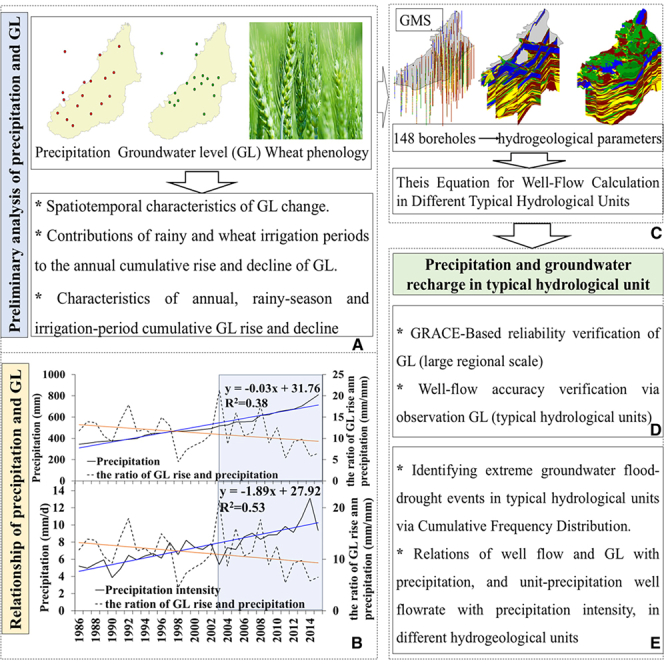

## Introduction

Groundwater is the primary water source for irrigating crops in global semi-arid agricultural regions, accounting for over 70% of water consumption.^1^^,^^2^ Sufficient groundwater recharge is critical to agricultural water resource management and constitutes a key safeguard for food security.^3^^,^^4^ Precipitation accounts for 30%–50% of total groundwater recharge, respectively—typical proportions reported in semi-arid agricultural zones 5,6—and is the most important factor affecting groundwater level (GL). In recent years, with the increased frequency of extreme precipitation, the groundwater recharge pattern has changed^5^; notably, intense but sporadic precipitation events often lead to surface runoff rather than infiltration, potentially decreasing the recharge rate.^6^^,^^7^ Currently, there remains a lack of research that quantifies the impacts of precipitation pattern variations on groundwater recharge based on long-term observed data.

Spatially, fluctuations in GLs are also influenced by hydrogeological conditions.^8^^,^^9^ The porous media of aquifer systems in piedmont areas (i.e., transition zones between mountains and plains) have high porosity and water content, which decrease as moving toward inland regions and reach the lowest value in lacustrine plains (plains formed by the deposition of sediments in ancient lake basins).^7^ This spatial pattern of aquifer physical properties leads to a similar spatial distribution in the vulnerability of exploitable groundwater resources—piedmont areas have relatively larger exploitable groundwater volumes, while inland regions have smaller ones.^10^ Similarly, owing to the differential capacity to receive precipitation infiltration, extreme precipitation exerts spatially heterogeneous impacts on groundwater recharge across distinct hydrogeological units. As global climate change intensifies,^11^ sustainable regional groundwater management is in urgent need of an in-depth understanding of the variations in groundwater recharge induced by extreme precipitation under different hydrogeological conditions, a knowledge gap that remains unaddressed to date.

Most existing studies on the spatiotemporal variations and attribution of groundwater balance rely on hydrological models, such as MODFLOW, HYDRUS, and SWAT.^12^^,^^13^^,^^14^ These classical physically based hydrological models can accurately capture the spatiotemporal variations in GL at regional scales, while also clarifying the impacts of precipitation events.^15^ However, due to substantial parameter uncertainties, these models struggle to capture the effects of extreme precipitation on groundwater recharge under different hydrogeological conditions.^16^^,^^17^ There is an urgent need to conduct a study on groundwater recharge characteristics entirely based on the observed data (including precipitation, groundwater, and aquifer core lithology) to accurately characterize the complex impacts of recent climate changes on GL. Although GRACE products also belong to the observed data, their coarse spatial resolution limits their application at the point scale.^18^^,^^19^^,^^20^ They are only suitable for verifying the accuracy of multiple point-scale observation results at the macro scale.

Accurately quantifying point-scale groundwater recharge and discharge without relying on hydrological models remains a key challenge in conducting groundwater balance analyses. The Theis equation plays a central role in quantitatively describing how GL (hydraulic heads) vary with time and distance in an aquifer during constant-rate pumping, serving as a fundamental formula for modeling transient groundwater flow in hydrogeology.^21^ Given known hydrogeological parameters and hydraulic head variations, well pumping rate dynamics can be inversely estimated, which in turn reflects groundwater storage changes—a method not yet put into practical application. The North China Plain (NCP), one of the world’s most representative semi-arid agricultural regions, has suffered severe groundwater depletion due to decades of over-irrigation, attracting widespread attention from governments and the global community alike.^22^^,^^23^ Although researchers such as Long et al.,^24^^,^^25^ and Zhang et al.^26^ have conducted extensive studies in this region, the influence of precipitation pattern changes on groundwater recharge rates remains unclear. There is an urgent need to apply the Theis equation to investigate the long-term dynamic response of GL to extreme precipitation.

**Figure 1:**
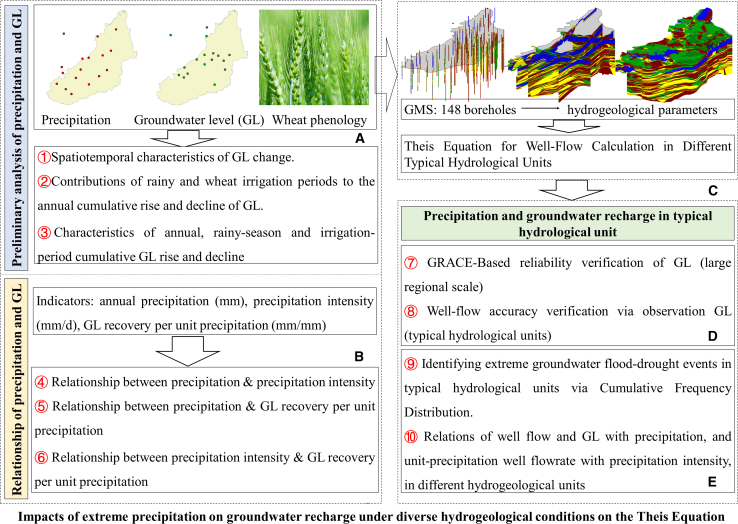
Schematic diagram of the research framework

Taking the plain of Baiyangdian Basin as a case, we innovatively propose a framework to evaluate the impact of extreme precipitation on groundwater recharge across distinct hydrogeological units (Figure 1). First, we preliminarily analyzed GL and precipitation data to characterize the spatiotemporal variations in GL, quantify the contributions of rainy seasons and wheat irrigation periods to annual cumulative GL fluctuations, and identify the cumulative GL change characteristics at annual, rainy-season, and irrigation-period scales (Figure 1A). Second, we quantified three key metrics—annual precipitation (mm), precipitation intensity (mm/d), and GL recovery per unit precipitation (mm/mm)—to clarify the relationships between precipitation and precipitation intensity, precipitation and unit precipitation-induced GL recovery, and precipitation intensity and unit precipitation-induced GL recovery (Figure 1B). Subsequently, based on the hydrogeological drilling data from 148 boreholes, we constructed a hydrogeological structure model, delineated typical hydrogeological units, and calculated the well-flow within each unit using the Theis Equation (Figure 1C). We used GRACE data to verify whether GL from observation wells in the 5 selected typical hydrogeological units could represent regional groundwater storage variations, and further validated the accuracy of groundwater flow-rate calculations using these 5 validated wells (Figure 1D). Via the cumulative frequency distribution method, we identified extreme groundwater flood-drought events across typical hydrogeological units. Finally, we quantified the relationships of well-flow and GL with precipitation, and unit-precipitation well-flow with precipitation intensity, in distinct hydrogeological units (Figure 1E). Through the above work, we accurately characterized the variation features and driving factors of GL and revealed the impacts of extreme precipitation on groundwater recharge. This study deepens the understanding of precipitation-groundwater hydrological processes and provides a theoretical basis for the spatial management of water resource exploitation under climate change.

### Study area

**Figure 2:**
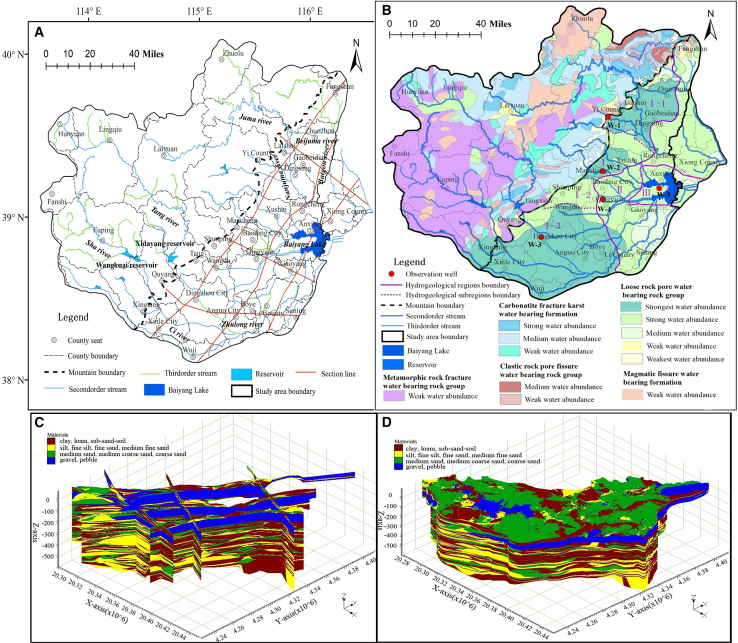
Hydrogeological settings and monitoring network in the Baiyangdian Basin (A) Surface water systems in the plain and mountainous areas. (B) Water abundance zones and lithological groupings. (C and D) Aquifer lithology (hydrogeological structure) in the plain area. The red line in Figure 2A indicates the locations of the six hydrogeological cross-sections shown in Figure 2C, while the red dots in Figure 2B denote the positions of groundwater monitoring wells within five typical hydrogeological units extending from the piedmont area to the lacustrine plain.

Baiyangdian Watershed (38°05′–40°09′N, 113°39′–116°18′E; Figure 2A) is one of the nine major sub-catchments of the Hai River Basin, covering an area of 36,000 km^2^ with a population exceeding 12 million.^27^ Characterized by a semi-humid climate, the region experiences an average annual temperature of 7.3°C–12.7°C and receives about 528 mm of precipitation per year. Precipitation is highly uneven, with 70–80% occurring as torrential rain between June and August.^28^ Since the 1960s–70s, over 1,400 reservoirs have been constructed in the upstream mountainous regions, controlling more than 90% of the basin area.^3^ Climate warming, reduced precipitation, and reservoir operations have decreased downstream flows, leading to perennial river desiccation and seasonal flow regimes; thus, groundwater in plain areas is almost entirely reliant on precipitation recharge.^1^^,^^29^ Within the same region, different precipitation intensities yield distinct groundwater recharge rates; similarly, an identical precipitation pattern produces varying recharge rates across different hydrogeological conditions.^6^^,^^30^ Given the increasing frequency of extreme climate events, there is an urgent need to quantify the impact of extreme precipitation on groundwater recharge under variable hydrogeological settings, thereby providing a scientific basis for accurate assessment of regional groundwater availability.

The study area was divided into mountainous and plain regions along the topographic boundary (Figure 2A). Mountains cover 61.7% of the total area, while plains account for 38.3%. Since the 1980s–90s, expanded agricultural land and intensified farming activities in upstream mountainous areas have led to groundwater over-exploitation, exacerbating water scarcity downstream.^29^ In the plains, groundwater over-extraction has formed funnel zones centered on Yimuquan. Gaoyang–Lixian–Qingyuan, and Baoding City,^31^ altering natural flow patterns by drawing surrounding groundwater toward these centers. Previous studies have confirmed that increased agricultural water use has intensified groundwater exploitation and induced GL decline. However, whether rising extreme precipitation frequency reduces groundwater recharge and further exacerbates this decline remains unclear.

## Results and discussion

### The spatiotemporal features of groundwater level change

#### Variations in groundwater levels of observation wells

The rise and decline of GL are direct indicators for evaluating the dynamic equilibrium of groundwater. The cumulative sums of monthly GL rise and decline in a year can effectively reflect the total recharge and discharge, respectively. The annual extreme range of GL can characterize the maximum annual groundwater withdrawal volume. Here, the three aforementioned indicators were employed to analyze the spatiotemporal variation trends and fluctuation amplitudes of GL. The nonparametric Mann-Kendall test was adopted to calculate the trend slope (k) and *p*-value, so as to identify whether the GL variation trends at each station represented genuine trends rather than random fluctuations. Significance was labeled as follows: ∗∗*p* < 0.001, ∗*p* < 0.01, and *p* < 0.05, and ns (non-significant, *p* ≥ 0.05). Additionally, the global Moran’s I test was conducted with an inverse distance spatial weight matrix constructed based on the actual latitude and longitude of 15 counties/districts, to examine the overall spatial distribution pattern (clustered or dispersed).

**Figure 3:**
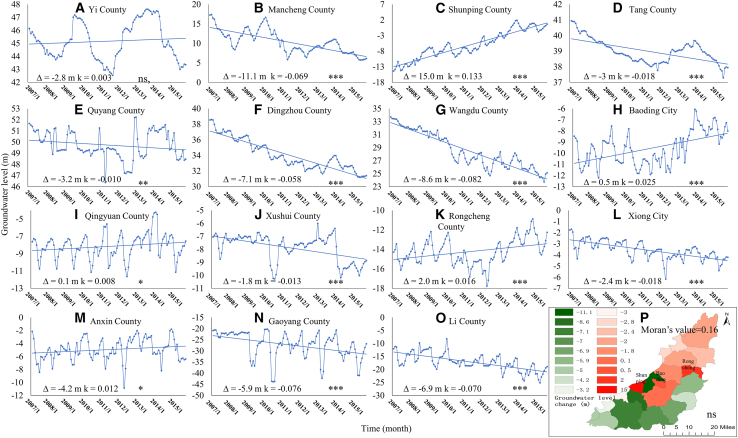
Monthly groundwater level fluctuations and spatial changes across counties from 2007 to 2015 (A–O) Monthly scale GL fluctuations. The vertical axis is the elevation of GL based on GPS observation. (P) Initial-to-final GL change distributions across counties. The symbol Δ is defined as the variation in GL between the initial period and the final period of the study. k is the trend slope. Significance was labeled as follows: ∗∗∗*p* < 0.001, ∗∗*p* < 0.01, and ∗*p* < 0.05, and ns (non-significant, *p* ≥ 0.05).

Compared with the initial study period, the GL increased significantly only in Shunping (+15 m, k = 0.133, ∗∗∗), Rongcheng (+2 m, k = 0.016, ∗∗∗), and Baoding Counties (+0.5 m, k = 0.025, ∗∗∗), whereas non-significant increasing or decreasing trends were detected in the remaining 80% of counties (Figures 3A–3P). The interannual fluctuations in GL basically presented a dynamic equilibrium state in four counties, namely Yixian, Quyang, Qingyuan, and Gaoyang (Figures 3A, 3E, 3I, and 3M). For the majority of observation wells exhibiting GL decline, the downward trend started to slow down around 2010, with a recovery trend observed in individual wells (Figures 3A, 3B, 3D, 3F–3K for details).

Spatially, the global Moran’s I was 0.16 with a *p*-value of 0.642 (ns), indicating no significant clustered or dispersed spatial distribution pattern of GL across the 15 counties/districts of Baoding City. The overall spatial correlation was weak, and the spatial variations in GL changes were mainly driven by local factors (e.g., local groundwater exploitation and topography). The degree of GL decline increases progressively from the piedmont regions to the inland regions (Figure 3P). For instance, the GL dropped by only 1.8 m in Xushui (Figure 3J), whereas it decreased by 6.9 m in Lixian (Figure 3O). This phenomenon is attributable to the abundant surface water resources and strong groundwater storage capacity in the piedmont regions (Figures 2A and 2B), which result in a relatively small GL decline in these areas under the same groundwater extraction intensity. The underlying cause lies in the differences in hydrogeological conditions.^32^ In addition, it is also associated with the spatial pattern of agricultural water consumption. Ma et al.^1^ showed that the north-to-south increasing trend of irrigation area and volume largely controls the GL pattern of “lower in the south and higher in the north.” Additionally, the northeastern part of the study area has abundant water systems (e.g., Juma River and Chaobai River; Figure 2A) and water inflow from upstream mountainous areas,^33^ resulting in insignificant GL decline there, whereas the opposite is true for the southwestern part. However, existing studies have failed to further reveal the mechanism by which the precipitation recharge rate in piedmont regions may be higher than that in eastern regions, thereby endowing piedmont regions with greater water resource carrying capacity.

#### Features of annual cumulative GL recovery and decline

**Figure 4:**
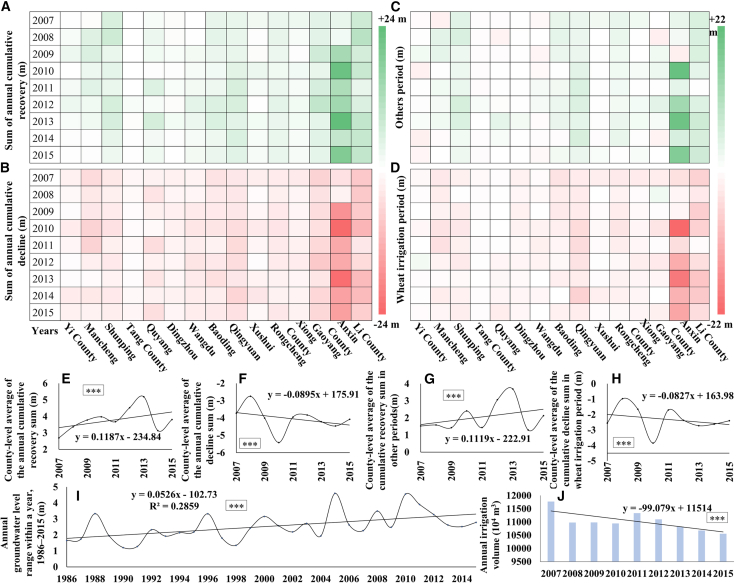
Cumulative monthly GL rise and decline during annual, non-irrigation, and irrigation periods, along with irrigation amount (A and B) Sum of monthly GL rise and decline values of observation wells in each county during 2007–2015. (C and D) Sum of GL rise values during the non-wheat irrigation period and the irrigation period of observation wells. (E and F) Mean value of the cumulative rise and decline values across all counties in panels (A) and (B), respectively. (G and H) Mean value of the cumulative rise and decline values across all counties in panels (C) and (D), respectively. (I) Annual difference between the maximum and minimum GL during 1986–2015. (J) Annual irrigation amount based on the statistical yearbook. Significance was labeled as follows: ∗∗∗*p* < 0.001, ∗∗*p* < 0.01, and ∗*p* < 0.05, and ns (non-significant, *p* ≥ 0.05). k is the trend slope.

In semi-arid regions, GL fluctuations are generally dominated by precipitation infiltration and irrigation. The magnitude of GL rise can reflect the impact of precipitation variability on groundwater and indirectly indicate precipitation effects on irrigation. Monthly GL rise and decline magnitudes were accumulated annually to decouple the characteristics of annual GL fluctuations (Figures 4A and 4B). This method was further applied to the non-irrigation period of wheat (mostly coinciding with the rainy season) and irrigation period (mostly coinciding with the dry season) to investigate the effects of precipitation and irrigation on GL (Figures 4C and 4D). In addition, county-level averages of the annual cumulative GL rise and decline values were calculated (Figures 4E and 4F), with separate computations also performed for the non-irrigation and irrigation periods (Figures 4G and 4H). Here, we only used GL changes during the irrigation period to identify potential drivers of GL decline and explore the possible impacts of precipitation intensity variability on irrigation. Our core focus lies on the effects of precipitation variability on GL rise, rather than quantifying the impacts of irrigation on groundwater resources—a complex scientific issue that warrants separate investigation.

We find that the wheat season (November to June of the following year) is the main period of groundwater decline, with an average decline of 2.3 m/year, accounting for 57.5% of the total annual groundwater decline (4.0 m/year on average) (Figures 4F and 4H). The non-wheat season (precipitation season) exhibits the opposite pattern (Figures 4E and 4G), contributing 55.3% to GL recovery. This indicates that GL fluctuations are dominated by precipitation and irrigation, consistent with the findings of Hu et al.^34^ This consistency corroborates the reliability of our data and results. The interannual fluctuations in the mean cumulative GL rise and decline across all counties exhibited a year-by-year increasing and decreasing trend, respectively (Figures 4E and 4F). This indicates that the interannual fluctuation of precipitation has become increasingly unstable, with a higher frequency of alternating extreme drought and extreme wet years. This interannual variability was more pronounced in the non-irrigation and irrigation seasons (Figures 4G and 4H), further indicating amplified interannual fluctuations in precipitation and an increasing frequency of flood-drought alternations. Notably, GL decline during the dry season has stabilized in recent years; even in the flood year (2013), excessive groundwater extraction remained considerable (Figures 4G and 4H). This demonstrates that elevated precipitation failed to reduce irrigation demand, corroborating that extreme heavy precipitation has a negligible effect on curbing agricultural water consumption.^5^^,^^35^ On a longer timescale, the intra-annual GL range exhibited an increasing trend (3-year moving average: 2.2 m in 1986 vs. 2.6 m in 2015) but declined after 2010 (Figure 4I), driven primarily by increased precipitation and reduced irrigation (Figure 4J).

Spatially, counties with larger cumulative GL rises also exhibited greater cumulative declines (Figures 4A–4D). The coefficient of determination (R^2^) of this relationship reached as high as 0.96. In the eastern region, the multi-year average cumulative rise values of Gaoyang, Li County, and Anxin were 13.6, 7.86, and 5.39 m/year, respectively, while their mean cumulative decline values were −14.19, −8.47, and −5.83 m/year. In the central region, the mean cumulative rise and decline values of Qingyuan, Baoding, and Rongcheng were 4.72 and −4.70 m/year, 3.66 and −3.52 m/year, and 3.27 and −3.01 m/year, respectively. In the western region (including Yi, Tang, and Dingzhou Counties), the corresponding multi-year averages were 1.29 and −1.56 m/year, 0.42 and −0.76 m/year, and 0.99 and −1.77 m/year, respectively. This spatial pattern indicates far more intense GL fluctuations in the east than in the west, likely driven by precipitation, irrigation, and hydrogeological conditions.^36^

In summary, the GL across the study area has exhibited a consistent declining trend over the long term, driven primarily by precipitation and irrigation. Over 80% of the counties have experienced a continuous long-term GL decline, with the rate of decline increasing progressively from the piedmont to inland regions. Increased precipitation and reduced irrigation have slowed the rate of GL decline. Cumulative GL recovery in the rainy season and decline in the irrigation period accounted for 55.3% and 57.5% of the annual total changes, respectively.

### Relationship of precipitation and groundwater level rise

**Figure 5:**
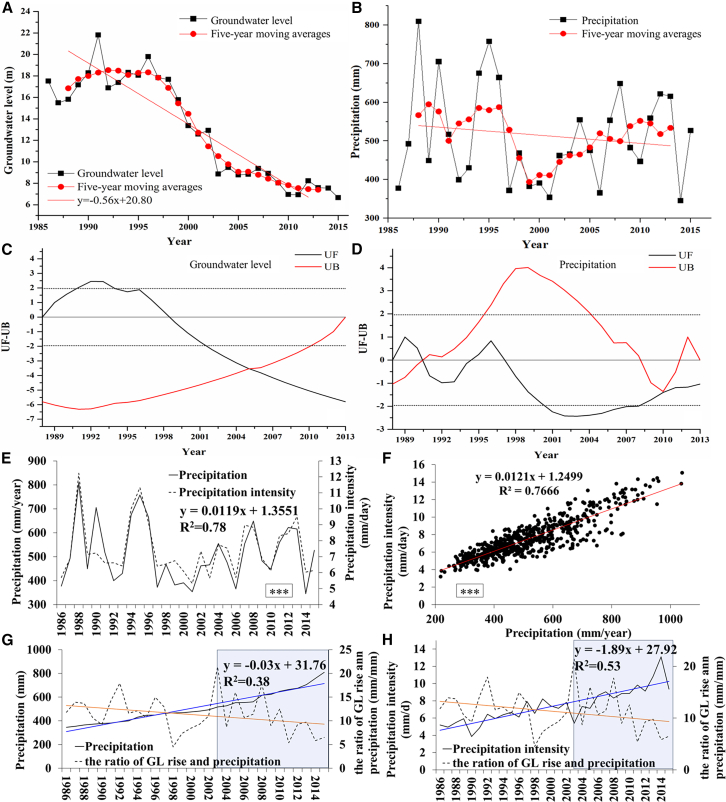
Interannual trends of GL and precipitation, and their relationships with precipitation intensity and GL recovery (A and B) Interannual variations in mean GL and precipitation across the study area. (C and D) Trends (Mann-Kendall) in mean GL and precipitation. (E and F) Relationships between precipitation and its intensity at regional and county scales. (G) Relationship between annual precipitation and GL recovery per unit precipitation. (H) Relationship between precipitation intensity and GL recovery per unit precipitation. Note: UB is used for comparison with UF; information such as change points in the time series is identified based on conditions, including their intersection. Typically, UF > 1.96 (α = 0.05) indicates a significant upward trend, UF < −1.96 a significant downward trend, and the intersection of UF and UB curves the candidate position of change points. Significance was labeled as follows: ∗∗∗*p* < 0.001, ∗∗*p* < 0.01, and ∗*p* < 0.05, and ns (non-significant, *p* ≥ 0.05).

Figures 3 and 4 have logically inferred that the amplified interannual fluctuations in GL and the increasing annual total GL recovery and decline magnitudes (i.e., groundwater flux) are both driven by extreme precipitation (i.e., elevated precipitation intensity). To further investigate the variations in precipitation and its intensity, as well as their impacts on GL recovery (i.e., groundwater recharge) over a 30-year long-term period, we conducted the analysis presented in Figure 5.

As shown in Figure 5A, the interannual variation of the average GL in the whole study area experienced three phases: a stable phase (before 1997), a rapid decline phase (1997–2005), and a slow decline phase (after 2005), with 2005 as the turning point (Figure 5C). The precipitation was approximately 700 mm/year before 1995, underwent a rapid decline during 1995–2000, and then showed an annual increasing trend thereafter, but never returned to the pre-1995 level (Figure 5B). The five-year moving averages (red lines in Figures 5A and 5B) eliminate uncertainties and better reflect the variation trends of GL and precipitation objectively. A comparison between Figures 5A and 5B reveals that although the interannual variations of precipitation and GL are not always consistent, precipitation generally exerts a strong control on groundwater fluctuations (R^2^ = 0.58, ∗∗∗). It should be emphasized that increases in annual precipitation are generally accompanied by elevated precipitation intensity, implying that longer precipitation durations do not correspond with greater precipitation amounts. Both at the regional and county scales (Figures 5E and 5F), precipitation and precipitation intensity exhibit an excellent correlation (R^2^ = 0.78 & 0.77, ∗∗∗ & ∗∗∗). This, however, may lead to a decrease in the groundwater recharge ratio.^37^^,^^38^ It can be observed that when annual precipitation exceeds 600 mm (e.g., 2003), the GL recovery per millimeter of precipitation decreases sharply (Figure 5G). Figure 5H more directly quantifies the relationship between precipitation intensity and GL recovery. When the extreme annual precipitation intensity exceeds 8 mm/day, the GL recovery per unit precipitation declines sharply (Figure 5H). Since 2003, driven by the increase in extreme precipitation, both precipitation and precipitation intensity exhibited a strong negative correlation with GL recovery (R^2^ = 0.38 & 0.53) (Figures 5G and 5H). This may have been overlooked in previous studies, leading to an overestimation of the recharge ratio of extreme precipitation to groundwater.^39^^,^^40^

In summary, the overall increase in total precipitation has been primarily driven by extreme heavy rainfall events. This has amplified both inter-annual and intra-annual fluctuations in GL, while simultaneously reducing the precipitation recharge rate. Even in wet years, GL still declines sharply during the irrigation period. When precipitation exceeds 600 mm/year (with intensity over 8 mm/day), the GL recovery and well-flowrate per unit precipitation both decline rapidly.

### Precipitation and groundwater recharge in a typical hydrological unit

#### The reliability of GL and Theis well flow rate

**Figure 6:**
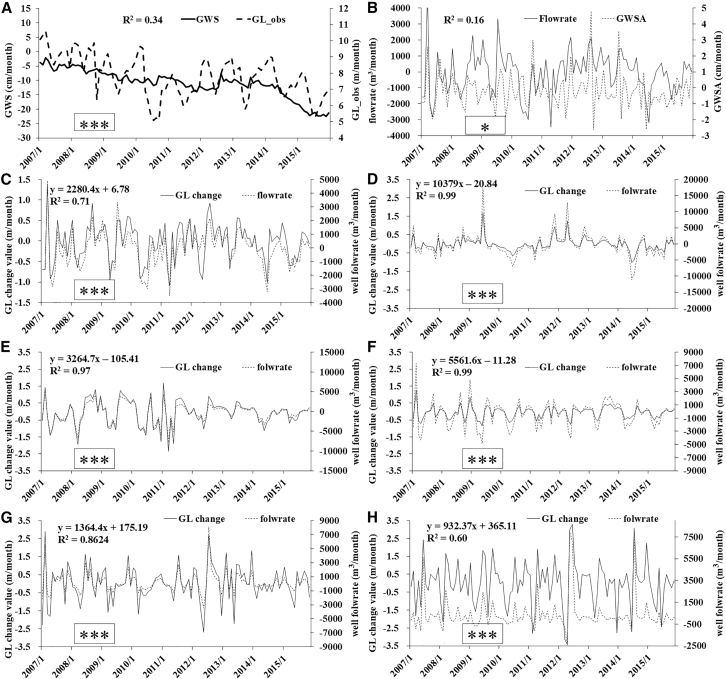
Validation of GL and well flow rate using GRACE data, and individual well relationships between ΔGL and flow rate (A) Relationships between groundwater storage (GWS) and the average GL of five typical wells. (B) Relationships between the average flow rate and groundwater storage anomaly (GWSA). (C) Relationships between the average GL and flow rate. (D–H) Relationships between ΔGL and well flow rate of individual wells. The D–H is in turn W-1, W-2, W-3, W-4, and W-5 in Figure 2B and Table 1. Significance was labeled as follows: ∗∗∗*p* < 0.001, ∗∗*p* < 0.01, and ∗*p* < 0.05, and ns (non-significant, *p* ≥ 0.05).

Considering the relative homogeneity of geological parameters within the same hydrogeological unit, we selected five GL observation points in Figure 2B to characterize the responses of regional GLs and well-flowrate to precipitation. First, we validated the accuracy of the regional GL (a state variable) and groundwater well-flow rate (a flux variable derived from GL fluctuations) using the groundwater storage (GWS) and its variations (GWSA), respectively, as retrieved from the GRACE data. This data is only used to verify the accuracy of GL and groundwater well discharge at the entire regional scale. As illustrated in Figures 6A and 6B, these variables exhibit highly consistent temporal trends, with corresponding coefficients of determination R^2^ of 0.34 and 0.16 (∗∗∗&∗), respectively. These validations demonstrate that the relationship between groundwater well flow rate and GL across the entire study area can be approximated using data from the five wells. On a monthly scale, a 1-meter rise in GL corresponds to an approximate increase of 2,287.2 m^3^ in groundwater well flow rate, with a coefficient of determination (R^2^) of 0.71 (∗∗∗) (Figure 6C).

Spatially, wells W-1, W-2, and W-3 are located in the piedmont area with relatively high μ_d_ and K (Table 1 and Figures 6D–6F). The changes in well-flowrate induced by GL fluctuation at these wells (y = 10379×, 3264.7×, 5561.6×, respectively) are significantly larger than those at Wells W-4 and W-5 (y = 1364.4×, 932.37×, respectively) (Figures 6G and 6H), which is consistent with theoretical expectations. The linear correlation between GL and well-flowrate is stronger for Wells W-1, W-2, and W-3 (R^2^ > 0.97, ∗∗∗) than for Wells W-4 and W-5 (R^2^ = 0.86 & 0.60, ∗∗∗&∗∗∗). This is also attributed to the higher hydrogeological parameter values in the piedmont area.

**Table 1:** The permeability coefficient K and specific yield μ_d_

	A, B, C		D, E, F, G		H, I, J		K, L		K	μ_d_
	Thickness	k	Thickness	k	Thickness	k	Thickness	k		
W-1	2	1.54	4	10.95	8	50	8	200	93.04	0.18
W-2	9	1.54	13	10.95	15	50	15	200	82.3	0.17
W-3	24.94	1.54	26.66	10.95	37.6	50	20.8	200	57.91	0.14
W-4	108	1.54	12	10.95	13	50	37	100	27.34	0.1
W-5	110	1.54	22	10.95	12	50	26.5	90	19.92	0.11

A, B, C, D, E, F, G, H, I, J, K, L respectively represent: clay, loam, sub-sand-soil, silt, fine silt, fine sand, medium fine sand, medium sand, medium coarse sand, coarse sand, gravel, pebble. k denotes the hydraulic conductivity of each lithologic layer, while the average hydraulic conductivity and specific yield of each hydrogeological unit are represented by K and μ_d_, respectively. The units of Thickness and k are “m” and “m/day,” respectively. W-1, W-2, W-3, W-4 and W-5 are groundwater level observation wells in five typical hydrogeological sub-units. Owing to consistent hydrogeological conditions in each sub-unit, their observed water levels can represent the average groundwater level variations of the respective sub-units.

#### The influence of precipitation on groundwater well-flow rate

**Figure 7:**
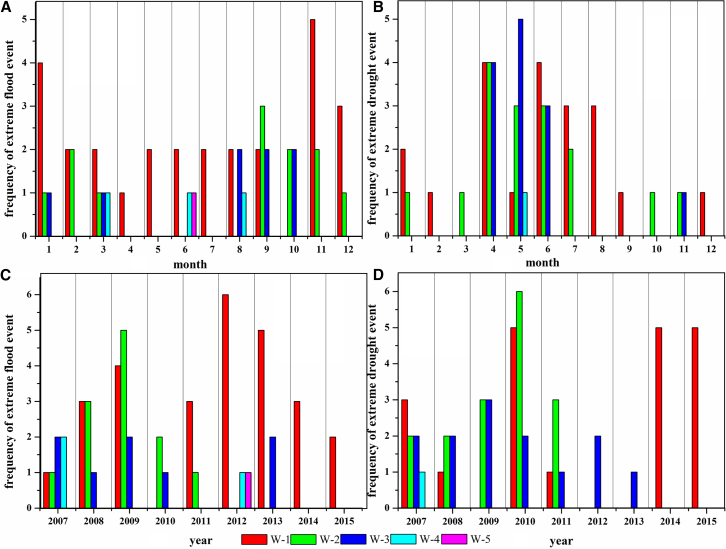
Extreme groundwater flood and drought events at five typical observation wells from 2007 to 2015 (A and B) Monthly scales. (C and D) Annual scales. Positive well flow-rate indicates flood events; negative well flow-rate indicates drought events.

As shown in Figures 7A and 7B, extreme flood events of positive well-flowrate mainly occur from September to December, while extreme drought events primarily happen from April to June (rain season in Figure 4G). This is strongly associated with the lag of precipitation recharge (precipitation generally occurs from July to October, with a lag period of approximately 1 month) and human activities of centralized groundwater exploitation for spring irrigation of wheat.^38^^,^^41^^,^^42^^,^^43^ The frequency of extreme flood and drought events in W-1, W-2, and W-3 is higher than that in W-4 and W-5, indicating that extreme precipitation exerts a weaker impact on groundwater recharge in piedmont regions (Figure 7). In these areas, the precipitation infiltration recharge rate is relatively high, enabling even short-term intense precipitation to effectively recharge the aquifers (Figure 6). Interannually, except for W-1 and W-3, the occurrence of extreme flood and drought events decreased markedly across all other regions during 2012–2015 (Figures 7C and 7D). This trend is strongly linked to the increase in extreme precipitation magnitude, the rise in precipitation intensity (Figures 5E–5H), and the reduction in irrigation volume (Figure 4J). Additionally, we observe that years with more frequent extreme flood events coincide with a higher occurrence of extreme drought events, which implies intensified groundwater abstraction concentrated in these years—primarily driven by irrigation demands. This finding aligns with the observation in Figure 4H that GL still experienced a substantial decline during wet years. Collectively, these results reflect that short-term extreme precipitation fails to alleviate irrigation water requirements; on the contrary, it necessitates greater groundwater extraction for irrigation purposes.

**Figure 8:**
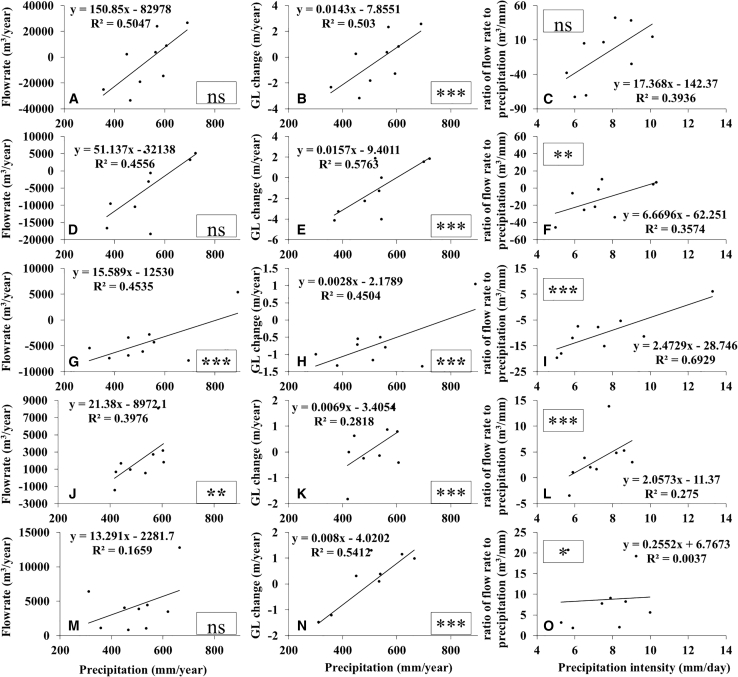
Relationships between precipitation, GL change, and well flow rate under different hydrogeological conditions (A–C) Relationships between annual groundwater well-flow rate and precipitation, GL change and precipitation, as well as well-flow rate per unit precipitation and precipitation intensity at Site W-1. (D–F) Relationships between annual groundwater well-flow rate and precipitation, GL change and precipitation, as well as well-flow rate per unit precipitation and precipitation intensity at Site W-2. (G–I) Relationships between annual groundwater well-flow rate and precipitation, GL change and precipitation, as well as well-flow rate per unit precipitation and precipitation intensity at Site W-3. (J–L) Relationships between annual groundwater well-flow rate and precipitation, GL change and precipitation, as well as well-flow rate per unit precipitation and precipitation intensity at Site W-4. (M–O) Relationships between annual groundwater well-flow rate and precipitation, GL change and precipitation, as well as well-flow rate per unit precipitation and precipitation intensity at Site W-5. Significance was labeled as follows: ∗∗∗*p* < 0.001, ∗∗*p* < 0.01, and ∗*p* < 0.05, and ns (non-significant, *p* ≥ 0.05).

Figure 8 more intuitively illustrates the effects of precipitation on groundwater well-flow rate and GL under diverse hydrogeological conditions. A comparison of Wells W1/W2 and W4/W5 (Figures 8A–8F, and 8J–8O) reveals that, owing to the higher specific yield and hydraulic conductivity in piedmont regions, precipitation induces a smaller rise in GL, a larger well-flowrate, and a weaker sensitivity of the flowrate-precipitation ratio to increased precipitation intensity, along with a stronger linear correlation. For instance, the slope values (k = 150.85, 51.14) and coefficients of determination (R^2^ = 0.50&0.46, ns&ns) of the linear equations for Figures 8A and 8D (W1, W2) are both higher than those for Figures 8J and 8M (k = 21.38, 13.29; R^2^ = 0.40, 0.17, ∗∗&ns). Consistently, the k and R^2^ values for Figures 8B and 8E exceed those for Figures 8K and 8N, and the R^2^ values for Figures 8C and 8F are higher than those for Figures 8L and 8O. Even when annual precipitation exceeds 600 mm (or daily precipitation intensity surpasses 8 mm/day), the linear relationships between precipitation and both well discharge and GL variations, as well as between precipitation intensity and the discharge-precipitation ratio, remain well preserved in piedmont regions (Figures 8A–8F, and 8J–8O). Nevertheless, it is worth noting that with an increase in extreme heavy precipitation events in the future, the recharge upper limit of piedmont regions may be reached, leading to a declining recharge rate and a heightened risk of surface flooding.^37^^,^^44^^,^^45^

In the eastern lacustrine plain area, the relationship between precipitation and groundwater well-flow rate is weak (Figures 8J–8O), indicating that groundwater recharge in this area is largely influenced by other factors. This is because the area is adjacent to the Baiyangdian Lake, with low topography (Figure 2A); factors such as surface water, lateral groundwater recharge, and farmland irrigation leakage all exert significant impacts on the variation of groundwater well discharge. In addition, the Middle Route of the South-to-North Water Diversion Project (SNWD) transferred a total of 23 million cubic meters of water to Baoding from January 27, 2015, to February 20, 2016 (https://www.baoding.gov.cn/content-173-94190.html). Compared with the groundwater extraction volume (Figure 4J), this amount is negligible. The imported water is insufficient to affect the overall groundwater changes across the region, but it may have a relatively significant impact on Well W5 (in Anxin County). This is because apart from meeting most of the domestic water demand, part of the SNWD water is used to replenish Baiyangdian Lake. However, considering the area’s thin aquifer (Figures 2C and 2D), small hydraulic conductivity, and low specific yield (Table 1), it can be reasonably inferred that the precipitation recharge rate to groundwater is inevitably much lower than that in the piedmont area, with a longer recharge lag period, thus resulting in higher vulnerability of groundwater supply.

In summary, due to higher permeability coefficients and specific storage capacities, piedmont regions are less vulnerable to extreme precipitation compared to inland areas. A strong positive correlation exists between high precipitation intensity and both GL and well-flowrate. These areas also experience more frequent extreme surges in well-flow rate, further confirming significant groundwater recharge.

### Limitations of the study

Precipitation is conventionally recognized as the primary driver of groundwater recharge in semi-arid areas.^23^^,^^46^ However, with the increasing frequency of extreme precipitation, its impacts on groundwater storage have become difficult to quantify.^42^ Different from traditional methods suitable for large scales—such as groundwater modeling and hydro-agrological-climatic coupled modeling—this study clarifies the spatiotemporal variation characteristics of GL at the observation point scale (Figures 3 and 4), reveals the controlling effects of precipitation, and hydrogeological conditions on GL changes (Figures 4 and 5), and identifies the annual precipitation thresholds causing abrupt changes in groundwater recharge across different spatial locations (Figures 6G and 6H). These results are based on abundant hydrogeological data, GL observation wells, daily precipitation data from meteorological stations, and wheat phenological data, combined with methods including the Mann-Kendall trend analysis, Theis Equation, and linear analysis. We provide a case demonstration and theoretical support for an in-depth understanding of groundwater change mechanisms and scientific water resource management in similar global regions. In addition, this study is entirely based on the observational data, avoiding parameter errors, interpolation errors, and systematic errors caused by spatial hydrological modeling. By selecting 5 observation wells representing typical areas according to hydrogeological zoning, it accurately reflects the patterns of precipitation impacts on groundwater changes across different spaces—a feature not available in other studies.^30^

However, it has a drawback of “using local observations to infer the overall situation,” which does not prevent us from using this method to gain a deeper understanding of the spatiotemporal evolution mechanism of groundwater and better address future water-food crises. In addition, this study relies heavily on the accuracy of observational data. The distances between GL monitoring wells, geological drilling cores, and meteorological observation stations must be relatively short to more accurately calculate the groundwater recharge and discharge flow rate at the observation points based on the Theis equation. This aspect also limits the application of this method in conducting intensive observation experiments on a large spatial scale.

## Resource availability

### Lead contact

Further information and requests for resources and materials should be directed to and will be fulfilled by the lead contact, Shumin Han (hansm@sjziam.ac.cn).

### Materials availability

This study did not generate new unique reagents or materials.

### Data and code availability

#### Data

The precipitation data, GL data, and hydrogeological drilling data used in this study are available from the corresponding author upon reasonable request. GRACE data (RL06) were obtained from NASA’s Goddard Space Flight Center (https://grace.jpl.nasa.gov/data/get-data/).

#### Code

No custom code was used in this study. The Theis equation calculations were implemented in Excel.

#### Other

Any additional information required to reanalyze the data reported in this paper is available from the lead contact upon request.

## Acknowledgments

This study was jointly supported by the Youth Top Talent Project of 10.13039/501100003482Hebei Provincial Department of Education (grant no. BJ2025106); the 10.13039/501100003787Natural Science Foundation of Hebei Province (grant no. D2025403081), the 10.13039/501100001809Natural Science Foundation of China (grant no. 42207551), the 10.13039/501100012166National Key R&D Program of China (grant nos. 2022YFB3903505 and 2022YFB3903005-4), the National Pre-research Project of 10.13039/501100008759Hebei GEO University (grant no. KY2024QN26), the 10.13039/501100001809National Natural Science Foundation of China (grant no. 42502257), and the 10.13039/501100017596Natural Science Basic Research Program of Shaanxi Province (grant no. 2025JC-YBQN-434).

## Author contributions

Q.M.: writing – original draft, conceptualization; X.Z.: methodology and data curation; Z.B.: visualization; S.P.: data curation and visualization; X.H.: data curation; Y.L.: data curation; G.S.: investigation; Z.C.: data curation; R.C.: investigation; Z.W.: data curation; S.H.∗: supervision and writing – review and editing; D.R.∗∗: writing – review and editing; X.L.: resources; Z.M.: methodology.

## Declaration of interests

The authors declare no competing interests.

## STAR★Methods

### Key resources table

**Table:** 

REAGENT or RESOURCE	SOURCE	IDENTIFIER
**Deposited data**		

GRACE monthly gravity field data (RL06)	NASA GSFC GRACE Data Distribution Portal	https://grace.jpl.nasa.gov/data/get-data/
Precipitation data (1986–2015)	Meteorological stations, China Meteorological Administration	Available upon request
Groundwater level data (1986–2015)	Local water resources departments, Hebei Province	Available upon request
Hydrogeological borehole data (148 cores)	Hebei Provincial Bureau of Geology and Mineral Resources Exploration and Development	Available upon request

**Software and algorithms**		

GMS (Groundwater Modeling System)	Aquaveo	https://www.aquaveo.com/software/gms
Excel	Microsoft	N/A

**Other**		

Theis equation calculation	This study	Custom Excel spreadsheet; available upon request

### Method details

#### Data collection and processing

To address these research needs, we compiled shallow groundwater hydrogeological maps (Figure 2B) using hydrogeological maps of Beijing, Hebei, Shanxi, and pumping well discharge data. To refine the spatial distribution of hydrogeological conditions, we mapped six hydrogeological profiles (Figures 2A and 2C) and constructed a hydrogeological structural model in GMS (Figure 2D), based on 148 hydrogeological core drilling datasets. According to lithological zoning and vadose zone structure, the plain area was divided into three broad regions, with Region I further subdivided into three subregions (Figure 2B). There have notable differences in water richness between the piedmont plain and mountainous areas, with generally greater water richness in the plains, indicating macro-topographic control over shallow groundwater resources. For example, the upper mountainous reach of the Sha River features a low-yield magmatic fissure aquifer, whereas the downstream area (I-3) is highly water-rich (Figure 2B). The middle area (II) and the eastern region near Baiyangdian (III) exhibit moderate and low water yield, respectively (Figure 2B), which is consistent with the spatial lithological distribution of the aquifers (Figures 2B–2D).

We established the hydrogeological structure of the plain area of the Baiyangdian Watershed using data from 148 borehole cores (Figures 1, 2C, and 2D). Lithologies were classified into clay, loam, sandy loam, fine silt, silt, fine sand, medium-fine sand, medium sand, medium-coarse sand, coarse sand, gravel, and pebble, and were generalized according to rock permeability. A water-richness zoning map was compiled based on existing hydrogeological maps and data, dividing the plain into three hydrogeological regions with one region further subdivided into three subregions (Figure 2B). Figure 2B is mutually corroborative and consistent with Figures 2C and 2D, which demonstrates the objectivity and reliability of the reported spatial variations in hydrogeological conditions and verifies the strong representativeness of the five selected typical hydrogeological zones.

To investigate the long-term (30-year) relationship between precipitation and GL, we collected precipitation station data (Figure 1) and GL monitoring records spanning 1986–2015. Data discontinuities existed at individual GL monitoring sites due to the commissioning of new wells and decommissioning of old ones; thus, we only used the compiled dataset to characterize the regional-average relationship between GL and precipitation. Prior to analysis, the reliability of the regional-average GL was verified using GRACE data. The GRACE satellite data used are the Level-3 monthly gravity field products (product version: RL06) jointly released by the National Aeronautics and Space Administration (NASA) and the German Aerospace Center (DLR). The data were acquired from NASA’s Goddard Space Flight Center (GSFC) GRACE Data Distribution Portal (https://grace.jpl.nasa.gov/data/get-data/). The original temporal resolutions were 1 month for GL data and 1 day for precipitation data, while GRACE data had a 1-month temporal resolution and 0.25° spatial resolution. The data time series is consistent with the observation period of GL, covering the complete period of groundwater dynamic monitoring in the study area. This relatively coarse spatial resolution had no impact on the present study, as GRACE data were solely used for validating regional-average GL reliability. A total of 16 wells with complete, continuous datasets were available for the period 2007–2015 (Figure 1); thus, these wells were selected to investigate the precipitation–GL and precipitation–well yield relationships at the site scale. Given the inconsistent spatial proximity between meteorological stations and groundwater wells, coupled with the uneven spatial distribution of station density, only one groundwater monitoring well and one precipitation station were selected per hydrogeological unit, on the basis of the uniform geological parameters within the same unit (Figure 2B). For data processing, the monthly mean values of the adjacent periods were used to fill in the gaps of missing GL data for individual months.

Precipitation intensity is defined as the precipitation amount per unit time, and its variability exerts a substantial impact on groundwater recharge rates.^47^ For a given annual total precipitation, recharge rates vary significantly across different precipitation regimes, namely short-duration heavy precipitation, long-duration heavy precipitation, and long-duration moderate-to-light precipitation.^38^^,^^48^ However, the complexity of field conditions—including antecedent soil moisture, soil type, groundwater level, post-precipitation evaporation, and groundwater extraction—obscures the quantitative relationship between unit precipitation and groundwater level rise.^49^^,^^50^ Disentangling the recharge effects of individual precipitation regimes is highly challenging and prone to large errors; these confounding factors can, nevertheless, be largely eliminated when extending the analysis to annual and monthly timescales. Given that this study focuses on how total precipitation and its intensity modulate groundwater recharge under heterogeneous hydrogeological conditions, rather than quantifying recharge from individual precipitation events, the analyses were conducted exclusively at annual and monthly scales. Annual precipitation intensity (mm/year) was calculated as the ratio of total annual precipitation to the number of rainy days, with monthly precipitation intensity derived using the same method.

#### Calculation of single-well flowrate via theis equation

The Theis equation serves as the core formula for unsteady groundwater flow calculations. Proposed by the American hydrologist Theis,^51^ it is designed to characterize the spatiotemporal evolution of drawdown induced by a single well pumping at a constant rate within a confined aquifer. The application of this equation is subject to the following assumptions: (1) The aquifer is homogeneous, anisotropic, and of uniform thickness, behaving as an elastic medium. (2) No vertical recharge or discharge occurs across the aquifer boundary. (3) Groundwater seepage conforms to Darcy’s law. (4) The well is fully penetrating, with radial flow uniformly converging toward the wellbore. (5) Groundwater release from storage in response to head decline is instantaneous. (6) The initial hydraulic head is uniformly distributed throughout the aquifer prior to pumping. (7) Pumping is performed at a constant discharge rate throughout the test period. 8) The aquifer extends infinitely in the lateral direction. If Assumption (1) is revised to “the aquifer is homogeneous, isotropic, of uniform thickness, and underlain by a horizontal impermeable base”, and a (9) assumption is added: “the magnitude of drawdown is much smaller than the thickness of the unconfined aquifer, and flow follows the Dupuit assumption”, the Theis equation can be extended to simulate flow toward a fully penetrating well in an unconfined aquifer.^52^ The corresponding governing differential equation is expressed as Equation 1. The model is described as follows:

(Equation 1) $$\begin{array}{c} {\text{Kh}}_{m}\left(\frac{\partial^{2}\varphi }{\partial r^{2}}+\frac{1}{r}\frac{\partial \varphi }{\partial r}\right)=\mu_{d}\frac{\partial \varphi }{\partial t}\;\left(0\leq r<\infty ,t>0\right)\\ \varphi =\frac{1}{2}h^{2}\;\\ \varphi \left(r,0\right)=\varphi_{0}\;\left(0\leq r<\infty \right)\;\\ \varphi \left(\infty ,t\right)=\varphi_{0}\;\left(t>0\right)\;\\ \lim_{r\to 0}2\pi \text{rK}\frac{\partial \varphi }{\partial r}=Q\;\left(t>0\right)\; \end{array}]$$

K: permeability coefficient (m/d); h: Hydraulic head of the unconfined aquifer (m); h_m_: Mean thickness of the unconfined aquifer (m); φ: Potential function (m^2^); φ_0_: Initial potential function value at the initial time, corresponding to the potential function of the initial hydraulic head (m^2^); r: Distance from observation well to pumping well (m); t: Pumping duration (d); μ_d_: Specific yield; Q: Constant pumping rate of the pumping well (m^3^/d).

Through a series of derivations and transformations, the fundamental equation for flow toward a fully penetrating well in an unconfined aquifer presented above can be expressed as Equation 2.

(Equation 2) $$Q=\frac{2\pi K\left(2h_{0}-s\right)s}{W\left(u\right)}$$

(Equation 3) $$W\left(u\right)=\int_{u}^{\infty }\frac{e^{-x}}{x}d_{x}$$

(Equation 4) $$u=\frac{r\mu_{d}}{4Kh_{m}t}=\frac{r^{2}}{4\text{at}}$$

(Equation 5) $$a=\frac{Kh_{m}}{\mu_{d}}$$

where s denotes the drawdown at the pumping well (m); W(u) represents the Theis well function; u is the dimensionless parameter for well flow; a refers to the aquifer head diffusion coefficient.

When the parameter u is sufficiently small, the Theis well function W(u) can be expressed by Equation 6; the constant pumping rate Q can be derived from Equation 7 (an approximation of the Theis equation, namely the Jacob equation).

(Equation 6) $$W\left(u\right)=\ln\frac{2.25\text{at}}{r^{2}}$$

(Equation 7) $$Q\approx \frac{2\pi K\left(2h_{0}-s\right)s}{\ln\frac{2.25\text{at}}{r^{2}}}$$

Based on the derivation of Formulas 1–7, it is theoretically feasible to calculate the groundwater flowrate changes within a certain range around observation wells by leveraging GL fluctuations, provided that hydrogeological parameters are known. Furthermore, for unconfined well flow calculations, the average aquifer thickness h_m_ can be expressed as $h_{0}-\frac{s}{2}$.^52^

In this study, the Baoding Plain, a fluvial-alluvial plain in the Haihe River Basin, satisfies the core prerequisites for the inverse calculation of the Theis formula, for the following reasons:(1) High degree of aquifer homogeneity: The unconfined aquifer in the Baoding Plain is dominated by Quaternary fluvial-alluvial sand and gravel layers, which are horizontally continuous and relatively homogeneous in lithology (Figures 2C and 2D). (2) Adaptability to simplified modification under small drawdown conditions: The thickness of the unconfined aquifer in the Baoding Plain ranges from 10 to 30 m, and the annual drawdown of conventional groundwater exploitation generally does not exceed 10 m (Figure 3), satisfying the requirement for small drawdown scenarios. (3) Favorable boundary conditions for formula application: Most areas of the Baoding Plain are free of distinct impermeable boundaries, making it suitable for calculating well flowrates using this formula. Furthermore, the objective of this study is not to rigorously and precisely control the application conditions of the Theis formula, but to utilize its functionality for single-well flowrate calculation to reflect the relationship between extreme precipitation and groundwater storage changes. Thus, we prioritize ensuring that the calculated results can accurately characterize the magnitude and trends of groundwater well flowrate variations under different hydrogeological conditions, rather than expending excessive effort on verifying the accuracy of inverse flowrate calculation. Given that the study area is a field site characterized by highly complex climates, hydrogeological conditions, and human activities, and the monitoring period spans 9 years, multiple influencing factors inevitably introduce certain calculation errors. However, these errors can be attenuated over the long-term observation timeline and therefore do not compromise the scientific validity of the conclusions.

It is proved by calculation that the head deceleration is the same in the range of $r_{q}\approx 0.2\sqrt{\text{at}}$.^52^ In this study, GL denotes monthly observational data (t = 30 days). Based on prior findings, hydraulic conductivity K ranges 20–100, aquifer thickness h_m_ 30–150 m, and dimensionless parameter u generally 0.1–0.2. The radial distance r is tentatively set at 100 m to calculate the groundwater well flow (Q) within this range. The specific yield and permeability coefficient refer to Qian’s research. Calculation of permeability coefficient by layering method where M_i_ is the thickness of aquifer i (Equation 8).

(Equation 8) $$K=\frac{K_{1}M_{1}+K_{2}M_{2}+K_{3}M_{3}+\ldots +K_{n}M_{n}}{M_{1}+M_{2}+M_{3}+\ldots +M_{n}}$$

To investigate the impact of extreme precipitation on groundwater discharge under varying hydrogeological conditions, we selected one GL observation well in each of the five typical hydrogeological units presented in Figure 2B. Based on the aquifer lithology data from Figures 2C and 2D, we calculated the mean hydrogeological parameters using Equation 8, and further estimated the monthly-scale well discharge via the Theis equation. The lithological properties (lithology, thickness) and hydrogeological parameters (K, μ_d_) of each typical hydrogeological unit required for calculating groundwater well flow via the Theis equation are detailed in Table 1. Spatially, the lithology within each hydrogeological unit can be regarded as homogeneous, which satisfies the applicability requirements of the Theis equation. The five selected typical observation sites are largely representative of the spatial variations in aquifer lithology across the entire study area. For example, alluvial fan areas near the outlets of the Sha, Tang, and Juma Rivers are dominated by gravel and pebble deposits with low sediment selectivity. A thick clay layer is present in the inter-fan area, with notably thick clay aquitards observed in Mancheng District. The aquifer in the piedmont region is relatively shallow, ranging from −50 to 100 m in thickness, underlain by bedrock. In the central plain (Area II, Figure 2B), sediment grain size decreases, layer thickness increases, and permeability is lower compared to the piedmont fan area. The central formations are well-sorted, with clearly alternating and thicker sequences of permeable and impermeable layers. In the Baiyangdian area and its surroundings, clay, silty clay, and silt dominate, characterized by fine particles and low hydraulic conductivity. Here, rock layers alternate frequently, and both aquifers and aquitards are thin. With reference to Figure 2B, variations in lithology are identified as the primary factor influencing the heterogeneity of groundwater enrichment. This underscores the controlling role of hydrogeological structure on groundwater recharge, discharge, and flow dynamics. The permeability coefficient (K) and specific yield (μ_d_) of the aquifers near the five observation wells are provided in Table 1.

#### Cumulative frequency distribution (CFD)

Extreme heavy precipitation is often accompanied by a short-term surge in groundwater recharge. To characterize the spatiotemporal features of groundwater recharge, we defined extreme recharge events on the basis of groundwater well-flowrate derived from the Theis equation. There are many methods to define extreme hydrological events, including PDSI,^53^ multivariate statistical analysis, precipitation anomaly percentage, and area weight method.^54^ Percentile threshold which widely used in international is a method to define extreme events from the perspective of probability statistics. This paper uses the cumulative frequency method to determine the threshold of extreme hydrological events.

The calculation methods are shown as follows:

(Equation 9) $$\text{CFD}=\frac{F_{i}}{F_{n}}\times 100\%\;\left(i=1,2,3\ldots ,n\right)$$

(Equation 10) $$F_{i}=\sum_{n=1}^{i}f_{i}$$

n: Number of numerical grades within the value range; f_i_: Frequency of variable occurrence of target event in the i-th numerical level; F_i_: Frequency of variable less than or equal to a certain upper limit value; CFD: Cumulative frequency distribution.

According to groundwater recharge and discharge, the positive value of Q is defined as a flood event. The Q values corresponding to the 10% CFD values are defined as extreme drought.^55^

#### Trend and mutation analysis

Mann-Kendall (M-K) is a method often used in meteorology to perform mutation tests.^56^ Here, the moving average was used to analyze the trend of precipitation and GL, and the MK method was used for mutation detection.^57^ The specific steps are as follows: Let the original time series be y_1_, y_2_, y_3_, …, y_n_, m_i_ represent the cumulative number of the i-th sample y_i_ greater than y_j_ (1 ≤ j ≤ i), and define the statistics with d_k_.

(Equation 11) $$d_{k}=\sum_{i=1}^{k}m_{i},\left(2\leq k\leq n\right)$$

Under the assumption that the original sequence is random and independent, the mean and variance of d_k_ are:

(Equation 12) E(d_k_) = k(k-1)/4

(Equation 13) var(d_k_) = k(k-1)(2k+5)/72

Normalizing the d_k_ of the above formula, we get:

(Equation 14) $${\text{UF}}_{k}=\frac{d_{k}-E\left(d_{k}\right)}{\sqrt{\text{var}\left(d_{k}\right)}}$$

UF_k_ composes a UF curve, and it can be determined whether there is a clear change trend by reliability test. This method is introduced into the inverse sequence, and another curve UB is calculated. The intersection point of the two curves within the confidence interval is determined as the mutation point. Given the significance level α = 0.05, the critical values of the statistics UF and UB are ±1.96. UF > 0 indicates that the sequence is on the rise; on the contrary, it indicates that it is on the downward trend, greater than or less than ±1.96, which indicates that the upward or downward trend is obvious.

### Quantification and statistical analysis

All statistical analyses were performed using Excel and R software (version 4.0.3). The Mann-Kendall trend test was used to determine the significance of groundwater level trends, with *p*-values <0.05 considered significant. The global Moran’s I test, employing an inverse distance spatial weight matrix, was applied to assess spatial clustering of GL changes. Linear regression analysis was used to quantify relationships between precipitation, GL recovery, and well flowrate (Figures 5, 6, and 8), with coefficients of determination (R^2^) reported. Extreme flood/drought events in well flowrate were identified using the 10th and 90th percentiles of the cumulative frequency distribution (Figure 7). Detailed statistical outcomes, including exact *p*-values and sample sizes, are provided in the respective figure legends and the Results section.

### Additional resources

No additional resources were used beyond those described above.
